# Inguinal Hernia Repair Under Local Anesthesia: Benefits, Contraindications, Barriers to Use; A Narrative Review

**DOI:** 10.1111/ans.70962

**Published:** 2026-09-22

**Authors:** Seyed Ahmad Masoumi Lari, Niloufar Roustaei

**Affiliations:** ^1^ Department of Biology York University, Farquharson Life Sciences Building Toronto Ontario Canada; ^2^ Independent Researcher Toronto Ontario Canada

**Keywords:** herniorrhaphy, inguinal hernia, local anesthesia, surgical barriers, tumescent anesthesia

## Abstract

**Background:**

Inguinal hernia repair is a common surgery worldwide. Despite the advantages of local anesthesia, many healthcare providers avoid using this method due to several barriers relating to patients, surgical team, and operating rooms.

**Method:**

A review of studies published in PubMed and Google Scholar was conducted to examine outcomes, costs, patient satisfaction, safety, and barriers associated with the use of local anesthesia for inguinal herniorrhaphy.

**Results:**

Local anesthesia was associated with many advantages including less postoperative pain, fewer complications, shorter hospital stay, lower healthcare costs, and safer in high‐risk patients compared with spinal and general anesthesia. New methods such as tumescent local anesthesia and the combination of hypnosis and conscious sedation with local anesthesia enhance the effectiveness and safety of local anesthesia, improving both surgeon and patient experience. Selected studies demonstrated that using local anesthesia in some traditional contraindications such as obesity and recurrent cases is a safe and effective option. Despite the strong evidence, there are some barriers that limit the use of local anesthesia including patient anxiety, intraoperative pain, surgeon discomfort, staff communication problems, logistic issues, and lack of institutional protocols.

**Conclusion:**

Local anesthesia is a safe and cost‐effective method for inguinal hernia repair. To facilitate the routine use of local anesthesia, it is crucial to address the barriers through training the surgical team, the development of standardized protocols and logistics, and enhanced patient awareness.

## Graphical abstract

This graphical abstract summarizes a narrative review examining inguinal hernia repair performed under local anesthesia (LA). It outlines the aim and methodology of the review, the key benefits of LA repair, its contraindications, and the barriers limiting its wider adoption. The review concludes that LA repair remains a safe and cost‐effective approach, especially in short‐stay settings and high‐risk patients.

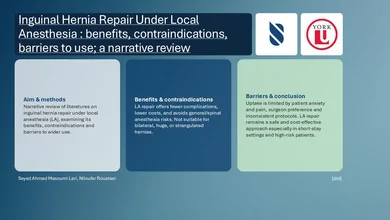

## 1 Introduction

Nowadays, healthcare systems all around the world face many challenges regarding patient satisfaction, costs, and quality of services. Healthcare providers continuously seek strategies to reduce costs, lower mortality and morbidity rates, and improve the quality of services to enhance patient satisfaction.

Inguinal hernia repair is a common surgery in the world that can be performed under general, spinal, or local anesthesia. Local anesthesia for inguinal hernia repair offers many advantages compared with general and spinal anesthesia such as reduced postoperative pain, earlier mobilization, shorter hospital stay, greater safety in high‐risk patients, fewer postoperative complications, and higher levels of patient satisfaction [1, 2, 3]. However, there are some contraindications for applying local anesthesia including huge, recurrent, strangulated, or bilateral hernia [1, 4]. Also, patients with certain underlying diseases, psychological problems, or known allergies to local anesthesia substances are not suitable candidates for local anesthesia [5, 6]. In recent years, advanced methods of local anesthesia have been used to improve both patient and surgeon experiences during the surgery.

Even though several studies recommend local anesthesia for the inguinal hernia repair, it remains underutilized in some countries [7]. Some factors contributing to its limited use include concerns about pain during the process, patient anxiety, lack of specialty surgical routines and proper logistics in surgical rooms, and challenges in team communication when patients are conscious.

The goal of this study is to demonstrate that the use of local anesthesia for inguinal hernia repair can reduce costs, decrease postoperative complications, and enhance both safety and patient satisfaction—factors which significantly impact on providing high‐quality healthcare services.

## 2 Methods

This narrative review was conducted using PubMed, OMNI search engine, and Google Scholar and covered articles published from January 2000 to March 2025. The keywords used to find the articles include “inguinal hernia,” “herniorrhaphy,” “hernia repair,” and “local anesthesia.” The studies were selected if they focused on inguinal hernia repair under local anesthesia with or without comparison to spinal or general anesthesia. The articles relevant to benefits, contraindications, barriers to utilization of inguinal herniorrhaphy under local anesthesia were screened. After excluding unrelated studies, 29 articles were included.

## 3 Results

The studies varied in design and patient population but collectively provide a comprehensive overview of the use of local anesthesia for inguinal hernia repair compared with other anesthesia methods. The key characteristics of the selected studies, including author and year of publication, study design, sample size, type of anesthesia used, hernia type, and the primary outcomes are presented in Table 1.

**TABLE 1:** Summary of included key studies on inguinal hernia repair under local anesthesia.

References	Design	Sample	Anesthesia type	Hernia type	Key outcomes/findings
Verma et al. [8]	Open‐label randomized controlled trial	60	Local vs. spinal	Unilateral inguinal hernia	Higher satisfaction under local anesthesia due to less pain and early mobilization
Gultekin et al. [9]	Prospective randomized	200	Local vs. spinal	Inguinal hernia	Lower hospital stay, no secondary infection, less material used under local anesthesia
Nordin et al. [10]	Randomized clinical trial	616	Local vs. spinal or general	Inguinal hernia	Local anesthesia has significant cost advantage over spinal and general anesthesia (*p* < 0.001)
Chyung et al. [6]	Retrospective	145	Local vs. spinal	Direct and indirect inguinal hernia	Tumescent solution is a safe and effective option for inguinal hernia repair
Romain et al. [11]	Prospective	103	Local with hypnosis vs. local and general	Inguinal hernia	Higher satisfaction and less pain at the hospital discharge (*p* < 0.001). Under local anesthesia with hypnosis
Leake et al. [12]	Randomized controlled trial	149	Local vs. local with conscious sedation	Inguinal hernia	Less pain and anxiety and higher satisfaction (*p* = 0.009) under combination of local and conscious sedation
Reid et al. [13]	Retrospective	125	Local	Inguinal hernia	Using local anesthesia is safe in obese patients
Gianetta et al. [14]	Retrospective	144	Local	Recurrent inguinal hernia	Local anesthesia is safe and effective method for recurrent inguinal hernia repair
Chen et al. [15]	Retrospective	90	Local vs. general	Incarcerated inguinal hernia	Practical to repair incarcerated inguinal hernia under local anesthesia
Ko et al. [16]	Retrospective	76	Local vs. general	Inguinal hernia	Operation time and total hospital stay are shorter in cases under local anesthesia
Olsen et al. [7]	Semistructured interviews	22	Local	Inguinal hernia	Barriers to using local anesthesia include feeling pain during surgery, difficult communication with staff, logistics problems, and lack of routine approaches

### 3.1 Benefits of Using Local Anesthesia for Inguinal Herniorrhaphy

Local anesthesia has been associated with several advantages over spinal and general anesthesia. For example, patients who are undergoing inguinal hernia repair under local anesthesia experience less pain after surgery due to longer duration of postoperative analgesia [1]. In addition, nausea and vomiting are less common after local anesthesia than general anesthesia [1, 17], and the incidence of urinary retention is significantly lower than that observed with spinal anesthesia [17, 18].

Patients undergoing herniorrhaphy with local anesthesia can mobilize and eat early after surgery, reducing their length of hospital stay as compared with other anesthetic techniques [19].

Utilizing local anesthesia decreases the total costs of the healthcare facilities through reducing use of operating rooms and postoperative care units [20].

One of the most important advantages of local anesthesia is that patients remain conscious during surgery, enabling them to perform the Valsalva maneuver while the surgeon confirms that the repair is complete and safe [21].

Some specific groups of patients may benefit from using local anesthesia including patients with cognitive dysfunction, the elderly, and patients with underlying disease who are at higher risk of postoperative complications under general anesthesia, such as cardiovascular and pulmonary dysfunctions [9, 21].

The use of local anesthesia solution facilitates dissection and helps the surgeon better identify the nerves and cord structure to avoid any serious tissue damage [22].

### 3.2 Contraindications of Local Anesthesia During Inguinal Herniorrhaphy

The reviewed studies recognized several absolute and relative contraindications of using local anesthesia to repair inguinal hernia. Absolute contraindications include hypersensitivity to lidocaine, bupivacaine, and other local anesthesia substances; uncontrolled cardiac arrhythmias; psychological dysfunction; coagulopathy; and huge, strangulated, or bilateral hernias [5, 6].

Relative contraindications reported in literatures include patients younger than 20 years, obese patients with BMI > 30, cases who are so nervous to endure being conscious during the surgery, patients who need other surgery combined with inguinal herniorrhaphy, suspected cases of strangulation, and patients with multiple recurrent inguinal hernia [5, 6].

### 3.3 Tumescent Local Anesthetic (TLA) Technique

Several studies described the application of tumescent local anesthesia for inguinal herniorrhaphy. That solution is a mixture of local anesthesia substances, epinephrine, sodium bicarbonate, and normal saline [23].

The injection of tumescent solution makes the dissection easier during the surgery because it provides a better view of the layers [6, 23]. Moreover, using epinephrine in the solution decreases the bleeding and prolongs the effect of local anesthesia [6, 24]. Adding sodium bicarbonate reduces the pain during the injection by buffering the acidic pH of lidocaine [25, 26].

### 3.4 Combination of Local Anesthesia With Other Methods

Some researchers reported the use of combined methods with local anesthesia to address patient anxiety during inguinal hernia repair. Hypnosis can modify pain perception, reduce patient anxiety, and even decrease pain at the time of discharge from hospital when combined with local anesthesia [11].

Another method that can decrease the patient's anxiety is conscious sedation, which is regularly used in same‐day surgeries [12].

### 3.5 Local Anesthesia Has Been Used for Relative Contraindicated Cases (Niche Applications)

Several studies demonstrated the use of local anesthesia in cases traditionally considered relative contraindications. These included overweight and obese patients, recurrent inguinal hernia repairs, emergency cases, and laparoscopic hernia repairs.

In obese patients, studies showed that local anesthesia is the better option to perform inguinal herniorrhaphy. However, concerns regarding the risk of using a high volume of local anesthesia were noted [26].

Inguinal hernia surgery under local anesthesia for recurrent cases was described as a low‐cost, safe, and effective method of treatment in selected cases [14].

Local anesthesia is considered a safe and practical option in emergency cases, as it reduces cardiopulmonary complications [15].

Laparoscopic total extraperitoneal (TEP) herniorrhaphy typically requires general anesthesia; however, one study demonstrated successful performance of laparoscopic TEP repair under local anesthesia in patients at high risk for general anesthesia [16].

### 3.6 Barriers to Using Local Anesthesia

The most important reason for avoiding local anesthesia is the probability of patients experiencing pain during the herniorrhaphy [1]. In addition, surgeons reported concerns regarding patients' anxiety related to being conscious during surgery [11] and intraoperative situations such as hypotension, sweating, or bradycardia [8].

Another concern arises from studies suggesting that the use of local anesthesia for inguinal hernia repair may increase the risk of hernia recurrence [15].

Some studies reported several factors contributing to underutilization of local anesthesia such as intraoperative pain, patients' anxiety, and difficult communication with staff when patients are awake. In addition, logistic problems in the operating room, lack of routine approaches, and surgeon discomfort have been mentioned as barriers [7, 27]. Surgeon experience and preference were identified as factors influencing the choice of anesthetic method [27, 28].

## 4 Discussion

This review identifies several economic and clinical advantages of using local anesthesia for inguinal hernia repair, while also highlighting its limitations and barriers to widespread adoption. Multiple studies show that local anesthesia is a safe approach compared with spinal and general anesthesia, especially in high‐risk patients and ambulatory surgical clinics. Across studies, local anesthesia was consistently associated with less postoperative pain, fewer complications—such as nausea, vomiting, and urinary retention—and a shorter hospital stay compared with general and spinal anesthesia. These benefits are related to the prolonged analgesic effect of local anesthesia and the absence of systemic effects associated with general and spinal anesthesia. In one study by Verma et al. [8] 65% of patients who had herniorrhaphy under local anesthesia reported higher levels of satisfaction due to less pain, early mobilization, and micturition compared with 37% under spinal anesthesia.

In addition, economic advantages were consistently reported. Several studies demonstrated significantly lower health care costs associated with local anesthesia due to decreased operating room time, shorter postoperative recovery and hospital stay, and reduced utilization of hospital resources. The study of Nordin et al. [10] demonstrated that costs of early postoperative hospital stay, the operating room, and postoperative care of local anesthesia are much lower than spinal and general anesthesia (*p* < 0.001) due to shorter time spent in these units. Gultekin et al. [9] stated the reasons for reducing costs using local anesthesia compared with general or spinal anesthesia. They realized that local anesthesia was associated with lower hospital stay, lack of secondary infections due to anesthesia, and less material used to apply the procedures [9].

These findings align with the global health priorities to provide high‐quality and cost‐effective healthcare services.

One remarkable benefit of local anesthesia is that patients are conscious, allowing them to perform the Valsalva maneuver to assess the safety of the repair during the surgery. Moreover, local anesthesia is a perfect option for elderly patients and those with underlying diseases or cognitive dysfunction due to elimination of the risks associated with general anesthesia.

This review also observed the inconsistencies regarding the role of local anesthesia in some relatively contraindicated cases such as recurrent hernia repair, emergency cases, obesity, and laparoscopic approaches. Some studies described using local anesthesia as a risk factor of hernia recurrence. However, findings across studies are inconsistent. The higher rate of recurrence was reported following repair of direct inguinal hernias, not indirect hernias and the recurrent rates for both direct and indirect hernia were found to be lower in private clinics compared with hospital settings [29]. Gianetta et al. [14] performed 144 anterior mesh repairs for recurrent inguinal hernia under local anesthesia with a 1.38% recurrence rate and a low complication rate (0.7%). That study demonstrates that inguinal hernia surgery under local anesthesia for recurrent cases can be a low‐cost, safe, and effective method of treatment.

Chen et al. [15] performed emergency inguinal hernia repairs in two groups—one under local and the other under general anesthesia—to evaluate the safety and effectiveness of using local anesthesia in emergency cases. The study showed that the rate of cardiac and respiratory complications is lower in the local group (*p* = 0.044, *p* = 0.027, respectively). Despite these findings, several studies emphasize that general anesthesia provides a more appropriate approach for the repair of huge, strangulated, or recurrent inguinal hernias because of the possible need for visceral exploration and altered anatomy [4]. As found by Reid et al. [13] obese patients do not need higher doses of local anesthesia, contrary to the main concern of surgeons who avoid using this method for obese patients.

Laparoscopic TEP herniorrhaphy is a low‐risk technique that is increasingly preferred by surgeons because it associates with less postoperative pain, faster recovery, and perfect cosmetic results [16]. However, this method requires general anesthesia to achieve muscle relaxation. Therefore, patients who are high‐risk candidates for general anesthesia, including elderly patients with coronary artery, cerebrovascular, or obstructive pulmonary diseases, should avoid TEP. Ko et al. [16] demonstrated that in high‐risk cases for general anesthesia, laparoscopic TEP repair under local anesthesia can be effectively conducted. Moreover, since patients are conscious during the surgery under local anesthesia, they can do the Valsalva maneuver to help the surgeon find the hernia sac. In addition, the nerve damage decreases due to the rapid response of patients when the nerve is touched.

Tumescent local anesthesia is an advanced technique that increases the safety and effectiveness of local anesthesia. In the study of Chyung et al. [6], they used the TLA solution (2% lidocaine 10 mL + 0.5% bupivacaine 10 mL + 0.1% epinephrine HCl 0.1 mL mixed with 80 mL normal saline made 100 mL TLA) for skin anesthesia and then injected it into the subcutaneous tissue and fascia layer. The diluted TLA solution provides effective intraoperative and prolonged postoperative analgesia for a wide area of skin and subcutaneous tissues with reduced risk of lidocaine toxicity compared with conventional local anesthesia. Also, this method provides easier tissue dissection by improving the visualization of anatomical planes, reduces intraoperative bleeding, prolongs the duration of anesthesia due to epinephrine, and decreases injection pain by the addition of sodium bicarbonate [6, 24, 25, 26]. These features may increase surgeon comfort and expand the applicability of local anesthesia in more complex cases.

Combining local anesthesia with methods such as hypnosis and conscious sedation can decrease patients' anxiety—one of the main factors affecting the choice of anesthesia—and improve overall patient experience. Romain et al. [11] studied the results of combining local anesthesia with hypnosis during inguinal hernia surgery and compared these outcomes with those of local anesthesia alone and general anesthesia. They identified that mean satisfaction scores are higher among the local anesthesia with or without hypnosis than general anesthesia (*p* = 0.07). Additionally, mean scores of preoperative anxiety were higher in the general anesthesia group compared with the other groups (*p* = 0.025). The mean scores of pain at the hospital discharge were lower among the local anesthesia with hypnosis than other groups (*p* < 0.001).

Leake et al. [12] performed a randomized trial study to evaluate the results of the combination of conscious sedation with local anesthesia compared with local anesthesia alone. They stated that adding conscious sedation to local anesthesia reduces patients' anxiety and pain feeling during the inguinal hernia repair and as a result increases patient satisfaction (*p* = 0.009) without prolonging the time of surgery or hospital stay.

Despite the numerous benefits of local anesthesia, many surgeons are reluctant to use this method. One qualitative study conducted by Olsen et al. [7] highlighted several concerns among surgeons when selecting local anesthesia over other anesthesia techniques. The most important concern was the possibility of causing intraoperative pain for patients that might compromise the surgeons' ability to optimally perform the procedure. Moreover, surgeons stated that communication with staff and residents becomes more challenging when the patient is awake, which could potentially increase patient anxiety, especially in complicated cases [7, 27].

Some surgeons also reported the absence of routine approaches for local anesthesia and facing logistical problems in surgical rooms where local anesthesia is not routinely practiced could make them avoid this method [7]. Nevertheless, Huerta [28] argues that the only barrier to the use of local anesthesia is surgeons' personal preference and other barriers can be overcome with training and experience.

This study is limited due to the lack of a systematic search strategy and formal quality assessment, which may cause selection bias. This review is mainly based on the existing research, which can be limited by some variables such as population, study designs, and results measures. In addition, some of the mentioned studies had small sample sizes or limited application to all settings due to differences in geographic and hospital practice. Therefore, additional randomized controlled trials (RCTs) and large‐scale observational studies are necessary to more conclusively assess long‐term outcomes, especially in relative contraindication scenarios.

Based on the reviewed studies, a unilateral and uncomplicated open herniorrhaphy can be safely performed under local anesthesia, especially in outpatient settings and in patients at high risk for general anesthesia. In addition, early mobilization, the ability to perform intraoperative Valsalva maneuvers, and decreased healthcare costs support its use as a preferred option for inguinal hernia repair.

Tumescent local anesthesia (TLA) provides long duration analgesia with reduced bleeding and injection pain and should be considered as an alternative to conventional local anesthesia. Adjunctive techniques such as sedation or hypnosis may also improve patient tolerance and satisfaction, increasing the feasibility of these methods in routine practice.

Despite supporting evidence about important benefits of local anesthesia for inguinal hernia repair, wider adoption needs to overcome systemic barriers. Surgical residents should receive standard training, and operating rooms should be improved by providing protocols and appropriate logistics for the administration of local anesthesia. Enhancing awareness among both patients and surgeons regarding its safety and efficacy may also help promote acceptance. Future research should focus on improving patient selection criteria, optimizing advanced techniques such as TLA, and exploring combined approaches to manage pain and anxiety, thereby providing a better experience for both patients and surgeons.

## 5 Conclusion

Inguinal hernia repair under local anesthesia is a safe and cost‐effective approach, especially in short‐stay settings and high‐risk patients. Advanced techniques such as tumescent local anesthesia and combination of local anesthesia with hypnosis or conscious sedation have been demonstrated to improve surgical outcomes and patient satisfaction. To enhance the utilization of local anesthesia, organized efforts should focus on training the residents and surgeons, establishing appropriate protocols and logistics, and increasing awareness among patients and healthcare professionals. Through these developments, local anesthesia could become a routine option for inguinal hernia repair, offering high‐quality health care services and improved patient satisfaction.

## Author Contributions

**Niloufar Roustaei:** conceptualization, data curation, writing – original draft. **Seyed Ahmad Masoumi Lari:** investigation, writing – review and editing.

## Funding

The authors have nothing to report.

## Disclosure

The corresponding author is not a recipient of a research scholarship.

## Conflicts of Interest

The authors declare no conflicts of interest.

## Data Availability

Data sharing not applicable to this article as no datasets were generated or analysed during the current study.
